# A hybrid molecular peapod of sp^2^- and sp^3^-nanocarbons enabling ultrafast terahertz rotations

**DOI:** 10.1038/s41467-021-25358-0

**Published:** 2021-08-25

**Authors:** Taisuke Matsuno, Seiya Terasaki, Kanako Kogashi, Ryosuke Katsuno, Hiroyuki Isobe

**Affiliations:** 1 Department of Chemistry, The University of Tokyo, Hongo 7-3-1, Bunkyo-ku, Tokyo 113-0033 Japan; 2 Department of Chemistry, Tohoku University, Aoba-ku, Sendai 980-8578 Japan

**Keywords:** Supramolecular chemistry, Molecular machines and motors, Carbon nanotubes and fullerenes

## Abstract

The internal hollow space of carbon nanotubes provides a unique nanometre-sized space to capture various molecular entities. The inner space circumfused by sp^2^-carbon networks can also encapsulate diamondoid molecules to afford sp^2^/sp^3^-hybrid nanocarbon peapods that have recently emerged as unique nanostructures. In this study, the sp^2^/sp^3^-hybrid peapods have been mimicked by adopting a cylindrical molecule and the smallest diamondoid, i.e., adamantane, to demonstrate the existence of ultrafast rotational motion. The solid-state rotational frequency is measured by NMR spectroscopy to record 1.06 THz that is, to the best of our knowledge, the largest value recorded for solid-state rotations of molecules. Theoretical calculations reveal that multivalent CH-π hydrogen bonds anchored the diamondoid guest on the π-wall of the cylindrical host. The weak hydrogen bonds are prone not only to cleave but also to regenerate at the interfaces, which give freedom to the guest for ultrafast isotropic rotations in the inertial regime.

## Abstract (web summary)

Mechanical motions in hybrid sp^2^/sp^3^ -hybrid nanocarbon peapods might lead to promising materials applications, but have been insufficiently explored. Here the authors demonstrate that a diamondoid molecule trapped inside a carbonaceous cylinder undergoes solid-state rotations at terahertz frequencies.

## Introduction

**Fig. 1:**
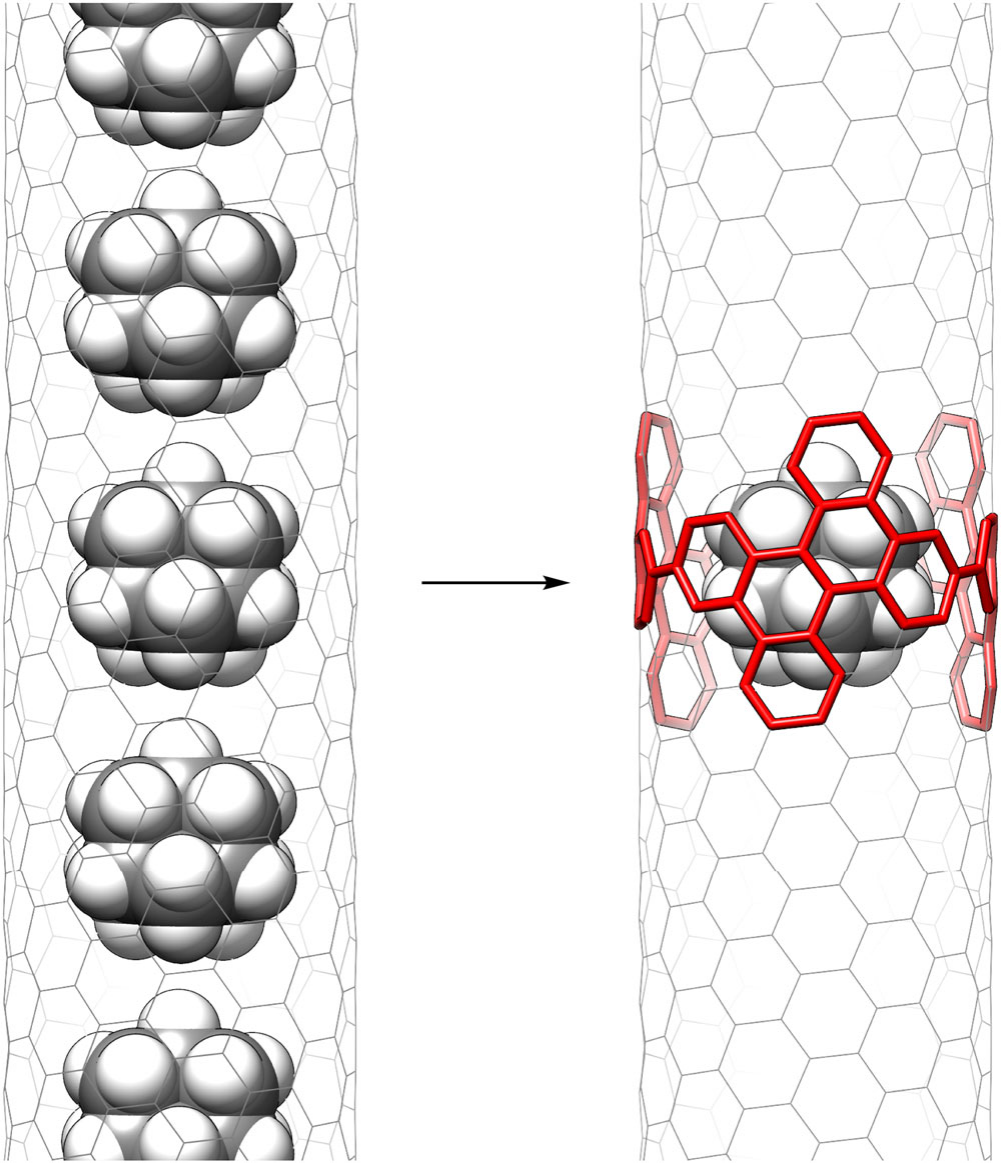
Hybrid nanocarbon peapods between sp^2^- and sp^3^ nanocarbons. A structure of adamantane molecules trapped in a (9,6)-helical CNT (left) and a segmental model molecule in this study (right) are shown.

Mechanical motions of nanometre-sized entities are expected to accompany unique physical properties^1^, and carbonaceous entities known as nanocarbons are attracting much attention^2–4^. Combinations of sp^2^-nanocarbons have been explored for nanoelectromechanic applications^5^, but hybrid combinations of sp^2^- and sp^3^ nanocarbons are much less exploited, despite the recent emergence of modern nanometre-sized sp^3^ diamondoids^6–8^. Although the sp^2^/sp^3^ hybrids of nanocarbons, such as nanocarbon peapods, have indeed been investigated both in theory and with experiments (Fig. 1)^9,10^, there exist contradictory proposals about the presence/absence of mechanical motion. For instance, experiments indicated that the smallest diamondoid molecule in CNTs is motionless and static^11^, but theoretical work predicted the existence of unique dynamic motion^12^. We envisage that a cylindrical molecule can serve as a segmental model of CNTs and that the dynamics of an encapsulated adamantane molecule may deepen our understanding of the sp^2^/sp^3^-hybrid peapods (Fig. 1). Here we show that a diamondoid molecule trapped in a circumfusing sp^2^ cylinder is given freedom for ultrafast, solid-state rotations at terahertz frequencies. Multivalent CH–π hydrogen bonds were prone not only to cleave but also to regenerate at the interfaces of nanocarbon hybrids.

## Results

### Assembly

**Fig. 2:**
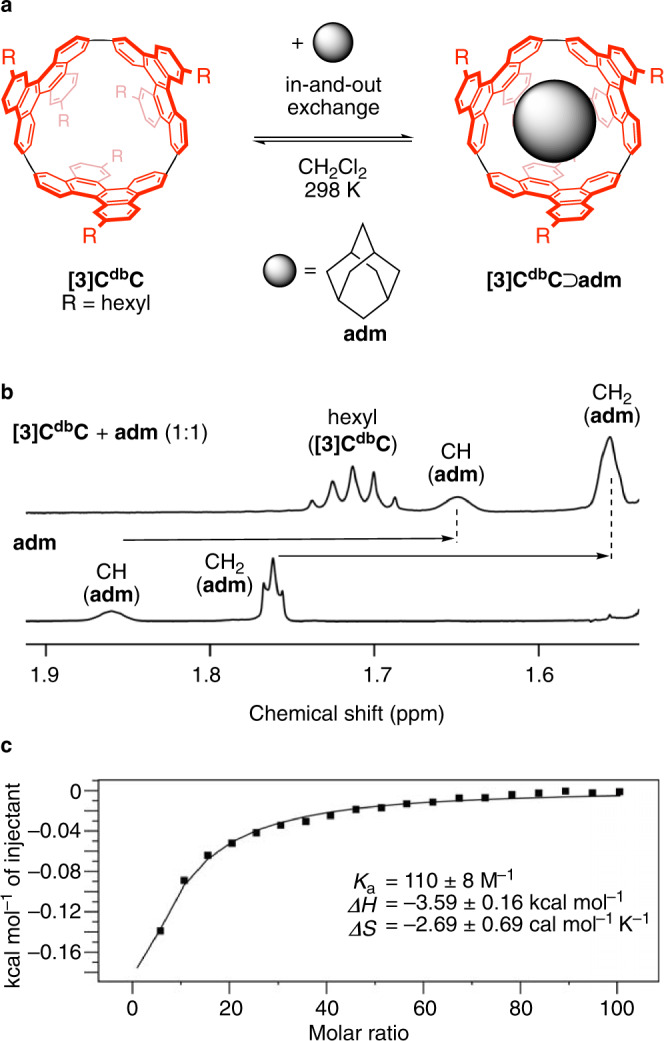
Assembly of a hybrid nanocarbon peapod in a molecular form. **a** Association equilibrium of the nanocarbon hybrid of **[3]C**^**db**^**C**⊃**adm**. **b** ^1^H NMR spectra of a mixture of **[3]C**^**db**^**C** + **adm** (1:1) and free **adm** in CD_2_Cl_2_ at 298 K showing high-field shifts of resonances of **adm**. A spectrum of the whole ^1^H region is shown in Supplementary Fig. 4. **c** Representative ITC data in CH_2_Cl_2_ at 298 K. Thermodynamic parameters are obtained from triplicate titrations (Supplementary Fig. 5), and the average values are shown with standard deviations.

The discrete molecular version of the hybrid nanocarbon peapod was assembled by using a cylindrical molecule, (*P*)-(9,6)-[3]cyclodibenzochrysenylene (**[3]C**^**db**^**C**)^13^, and the smallest diamondoid, i.e., adamantane (**adm**)^6^ (Fig. 2a). Encapsulation of **adm** in **[3]C**^**db**^**C** was first demonstrated by solution-phase NMR analyses. Upon mixing **adm** with **[3]C**^**db**^**C** in CD_2_Cl_2_, we observed upfield shifts of the ^1^H resonances of the guest. The induced shifts reached 0.2 ppm at 298 K for both methylene (CH_2_) and methine (CH) resonances (Fig. 2b), which corresponded well with a hybrid structure having the **adm** molecule trapped within the carbonaceous cylinder. When we recorded ^1^H NMR spectra of a 1:2 mixture of **[3]C**^**db**^**C** and **adm** while lowering the temperature, the resonances of **adm** coalesced at 203 K and split into two sets of resonances below this temperature (Supplementary Fig. 1). The observations showed that the **[3]C**^**db**^**C**⊃**adm** complex was under equilibrium conditions of rapid in-and-out exchanges that were retarded to the NMR timescale at 203 K. In a separate set of experiments, the association stoichiometry of 1:1 was determined by Job plot analysis (Supplementary Figs. 2 and 3), and isothermal titration calorimetry (ITC) analyses were performed under this condition (Fig. 2c)^14^. Considering the 1:1 stoichiometry, we then revealed the association thermodynamics with *K*_a_ = 110 ± 8 M^–1^ (Δ*G* = –2.79 ± 0.04 kcal mol^–1^; 298 K), Δ*H* = –3.59 ± 0.16 kcal mol^–1^, and Δ*S* = –2.69 ± 0.69 cal mol^–1^ K^–1^ via triplicate titration experiments. A complete picture of the energetics is summarised in Supplementary Fig. 6.

### Crystal structures

**Fig. 3:**
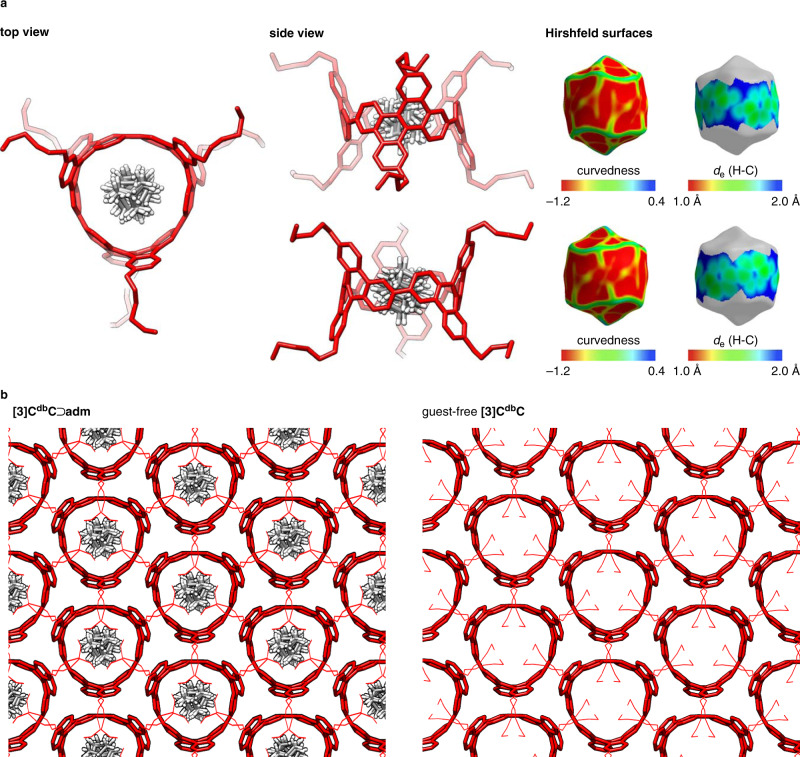
Crystal structures of a hybrid nanocarbon peapod. **a** Molecular structures and Hirshfeld surface analyses of encapsulated **adm** molecules. Solvent molecules, minor alkyl conformers, and hydrogen atoms of **[3]C**^**db**^**C** are omitted for clarity. Solvent molecules had no direct contact with the **adm** guest (see also Supplementary Fig. 11). The details of the disordered structures are shown in Supplementary Figs. 7 and 8. **b** Packing structures of **[3]C**^**db**^**C**⊃**adm** and guest-free **[3]C**^**db**^**C**. When the two panels shown in **b** are viewed as an autostereogram, one may easily notice that the crystal structures of cylinders are unaffected by the introduction of **adm** guests.

The molecular structure of the **[3]C**^**db**^**C**⊃**adm** hybrid was then determined by X-ray crystallographic analyses. As shown in Fig. 3a, the **[3]C**^**db**^**C** cylinder encapsulated the **adm** guest with an ideal peapod structure that matched well with TEM images of **adm** peapods formed with infinite CNTs^11^. Although the **adm** guest adopted six different orientations in the cylinder, the centre of mass of each orientation was approximately located at the centre of mass of the cylinder. By using the Hirshfeld surfaces of the **adm** guest as the probe^15^, the inner surface of the **[3]C**^**db**^**C** cylinder was further inspected. As was the case with larger, belt-persistent cylindrical molecules^16^, the arylene panels of the cylinder were smoothly curved, and inflection nodes of the curvedness surfaces did not segmentalise the inner surfaces of the cylinder. Contacts between carbon atoms of **[3]C**^**db**^**C** and hydrogen atoms of **adm** were also visualised by *d*_e_ mappings (Fig. 3a), which revealed the presence of CH–π contacts of **adm** with the belt-shaped wall of **[3]C**^**db**^**C**.

The crystal packings of **[3]C**^**db**^**C**⊃**adm** were also found to be unique. As we used a chiral (*P*)-isomer of the cylindrical host with *D*_3_ point symmetry throughout this study, the **[3]C**^**db**^**C**⊃**adm** hybrid was also chiral. The hybrid nanocarbon molecules were packed in a chiral crystal with a class-III, achiral space group of *P*321^17^. As shown in Fig. 3b, the cylindrical hosts were arrayed in a two-dimensional layer by forming hexagonal networks with two hexyl side chains pinching a wall of a neighbouring molecule, and the layers were stacked to form the chiral crystal (Supplementary Fig. 9 and 10). Within the two-dimensional layer, the nanocarbon hybrids were packed in a wallpaper plane group of *p*3 with multiple *C*_3_ symmetry axes standing along the cylinders^18,19^. Although we only obtained a single crystal of the (*P*)/(*M*)-racemate of the cylinder in a previous study^13^, we managed to grow a crystal of the guest-free (*P*)-cylinder for comparison in this study. Interestingly, the guest-free **[3]C**^**db**^**C** cylinders were arrayed in an identical structure with the *p*3 plane group to form a chiral crystal with the achiral *P*321 space group (Fig. 3b). The inner spaces of the cylinders were thus preassembled to form honeycomb pores before encapsulating the **adm** guest. Similar preassembly of cylindrical hosts was also observed with larger cylindrical hosts in previous studies^20,21^. The robustness of the crystal packings possessing the *C*_3_ symmetry axes with chiral fluorescent nanotubes is also of interest for exploration in future studies of materials applications^22^.

### Theoretical pictures of rotational motions

**Fig. 4:**
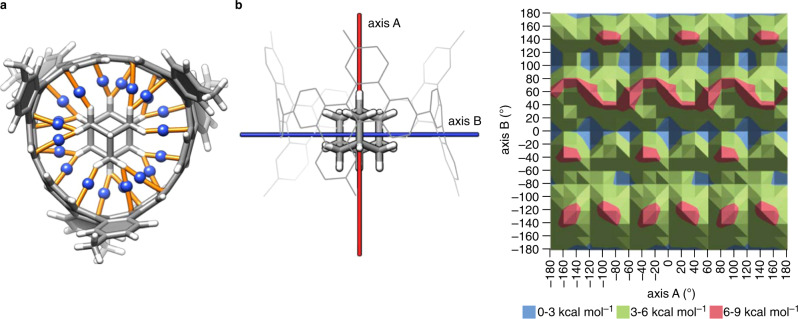
Theoretical pictures of hybrid nanocarbon peapods. **a** Hydrogen bonds at the interface of **[3]C**^**db**^**C**⊃**adm**. Bond paths (orange) and (3,–1) bond critical points (blue) from AIM analyses are shown. **b** Energy profile for the hypothetical rotations of **adm** within **[3]C**^**db**^**C**. The most stable global minimum is located at (A,B) = (0,0). See also Supplementary Movies 1 and 2.

Despite the unique structural details disclosed by the crystal structures, an important question about the static/dynamic behaviours of diamondoids within carbonaceous cylinders remains unanswered. We then started examining the possibility of rotational dynamics by performing theoretical calculations. By using the crystal molecular structure of **[3]C**^**db**^**C**⊃**adm** as the initial geometry, we performed geometry-optimisation calculations with density-functional theory (DFT) at the LCBLYP/6-311 G(d) level of theory, and the electron densities were further analysed with atoms-in-molecule (AIM) calculations^23,24^. As shown in Fig. 4a, the optimised structure reproduced the crystal structure with the encapsulated **adm** guest located within the cylinder. AIM analyses revealed the presence of multiple CH–π hydrogen bonds that anchored the guest on the wall of the cylindrical host^21,24–26^. Between the host and the guest, 21 bond paths appeared between the carbon and hydrogen atoms in total. To elucidate basic energetics for possible rotational motion, we estimated energy changes through hypothetical rotations of the **adm** guest within the static host by rotating the guest by 20° for single-point calculations. Two rotational axes were adopted: axis A is parallel to the cylinder axis, and axis B is perpendicular to axis A through the centre of mass of **adm**. The calculations resulted in the energy profile shown in Fig. 4b. The energy barriers for the rotations were so low, with Δ*E* < 9 kcal mol^–1^ viewed from the global minimum at (A,B) = (0,0). Two representative paths for one-turn 360° rotations were analysed in detail. Rotations from (A,B) = (–180,0) to (180,0) recorded a Δ*E* barrier of +3.17 kcal mol^–1^, and those from (A,B) = (0,–180) to (0,180) recorded a Δ*E* barrier of +7.16 kcal mol^–1^. As depicted in Supplementary Movies 1 and 2, the energy barriers originated from the cleavage of CH–π hydrogen bonds, but the multivalency achieved with the circumfusing π-wall for regenerating hydrogen bonds allowed for smooth rotations of the guest without forcing high-energy barriers. As characterised in the colour mapping images shown in Supplementary Fig. 12, CH–π hydrogen bonds were examined in detail for 5184 hydrogen atoms of 18×18 orientations of **adm**. The colour map showed that the number of hydrogen bonds depended on the locations of hydrogen atoms and varied from 3 to 0, but the regions circumfused with the cylinder were found to provide a hydrogen bonding continuum. The present theoretical analysis uses a very primitive, rough model, and further elaborate cutting-edge theoretical studies are desirable to disclose precise dynamic structures in the future. Nonetheless, this model nicely mimicked the crystal structure having the static cylinder with dynamically located **adm** molecules (Fig. 3a), showed possible connecting paths between various locations, and reproduced the energy barrier for the rotations (Δ*H*^‡^ = + 2.80 kcal mol^–1^; see below).

### Solid-state terahertz rotations

**Fig. 5:**
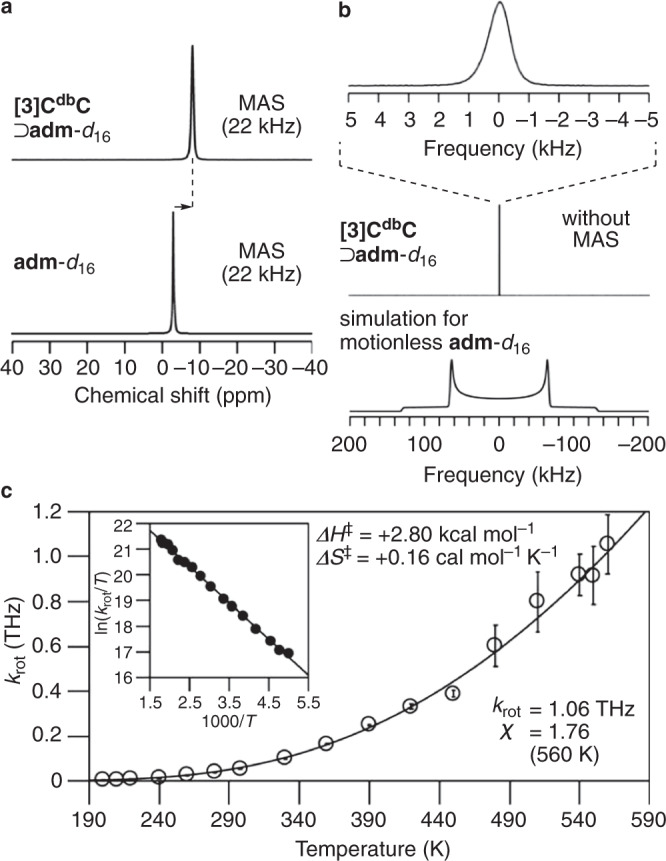
Ultrafast solid-state rotations of a diamondoid molecule in the hybrid nanocarbon peapod of adm⊃[3]C^db^C from ^2^H NMR analyses. **a** A high-field shift observed between the spectra of **[3]C**^**db**^**C**⊃**adm**-*d*_16_ and free **adm**-*d*_16_ under MAS conditions (298 K). **b** A symmetric narrow peak of **adm**-*d*_16_ observed with **[3]C**^**db**^**C**⊃**adm**-*d*_16_ under static conditions without MAS (298 K). A hypothetical spectrum simulated for motionless **adm**-*d*_16_ is shown at the bottom as a reference to show its absence. **c** Temperature-dependent rotational frequency (*k*_rot_) recorded with **[3]C**^**db**^**C**⊃**adm**-*d*_16_. The bars show the standard errors of the estimate.

Finally, the existence of ultrafast rotations of the diamondoid molecule within the carbonaceous cylinder was experimentally revealed by solid-state NMR analyses of **[3]C**^**db**^**C**⊃**adm**. Encapsulation of the deuterated **adm**-*d*_16_ guest within the **[3]C**^**db**^**C** host in a solid specimen was first confirmed by observations of an upfield shift of the ^2^H resonance (Fig. 5a) under magic-angle spinning (MAS) conditions. When MAS is turned off, a Pake doublet with a large coupling constant should normally be expected for a motionless, static **adm** guest as shown in the simulated spectrum (Fig. 5b). In an actual experiment with **[3]C**^**db**^**C**⊃**adm**, the 100-kHz Pake doublet was not observed, and, instead, a sharp symmetric peak with a half-width of 0.8 kHz was observed. This observation demonstrated the rapid rotational motion of the **adm** guest within the cylinder^27^.

Kinetics of the ultrafast, solid-state rotations of the **adm** guest in the cylinder were then revealed. The kinetics were investigated through measurements of the spin-lattice relaxation time (*T*_1_) of ^2^H. Thus, the *T*_1_ values were recorded by using a saturation-recovery method for the temperature range between 200 and 560 K (Supplementary Fig. 13). The *T*_1_ values were converted to the rotational correlation time (*τ*) and its reciprocal rotational frequency (*k*_rot_) by using Eq. 1 (see “Methods”). Starting at 4.61 GHz and 200 K, the *k*_rot_ values for the solid-state rotations of **adm** within **[3]C**^**db**^**C** increased with increasing temperature. At 560 K, the *k*_rot_ value reached the terahertz regime, i.e., 1.06 THz, which was, to the best of our knowledge, the largest *k*_rot_ value recorded for solid-state rotations of molecules. The rotational dynamics were further analysed by a method called the *χ* test via comparisons of the *τ*_FR_ values of ideal free rotations in the inertial regime (*χ* = *τ*/*τ*_FR_)^28^. The *χ* value of **adm** at 560 K was 1.76 and, as “*χ* = 2” is considered the threshold between diffusional rotations and inertial rotations, a 1.06-THz rotation of **adm** was proven to be achieved in the inertial regime^27^. The temperature dependency of the *k*_rot_ values also allowed us to reveal the kinetic barriers with the Eyring plot (Fig. 5c, inset). Thus, the enthalpy barrier (Δ*H*^‡^) for solid-state rotation was +2.80 kcal mol^–1^, which was close to the smallest theoretical *ΔE* value of +3.17 kcal mol^–1^ for the hypothetical 360° rotation (Fig. 4)^29^. The negligible Δ*S*^‡^ value of +0.16 cal mol^–1^ K^–1^ may correspond to the existence of multivalent CH–π hydrogen-bonding sites available for isotropic rotations (Fig. 4 and Supplementary Fig. 12).

## Discussion

In summary, a supramolecular combination of sp^2^- and sp^3^-nanocarbon hybrids was mimicked in the form of discrete molecular segments. Important roles of CH–π hydrogen bonds and their multivalency in the supramolecular assembly were disclosed both by experiments and theory. The π-wall circumfusing the hydrogen-terminated sp^3^-nanocarbon can provide an ideal anchoring continuum for association and dynamics. Although CH–π hydrogen bonds are inevitably directional^25^, their strength and availability of multiple binding sites allow smooth cleavage/regeneration for dynamic motion. The rotational motion of the smallest diamondoid molecule in a carbonaceous cylinder was indeed demonstrated by NMR spectroscopy, which provided conclusive evidence to support the existence of the dynamic motion of the sp^2^/sp^3^-hybrid nanocarbons. Interestingly, the ultrafast terahertz rotational frequency of the diamondoid molecule in the cylinder was spectroscopically demonstrated, and detailed analyses of the energetics of the rotations revealed that the interface of sp^2^/sp^3^ nanocarbons was as smooth as the interfaces of sp^2^/sp^2^ nanocarbons^27^. Given recent terahertz technology^30^, we hope that nanocarbon hybrids will be exploited as functional materials that could resonate at the terahertz frequency range via solid-state mechanical motion^31–33^. Along with unique rotational dynamics that reach the inertial regime, the terahertz rotations in the solid-state supramolecular assembly should be further explored to determine unique physical phenomena. The chirality of 2D-arrayed cylinders surrounded by multiple *C*_3_ axes could also lead to unique findings in material applications in the future.

## Methods

### Materials

The host molecule, **[3]C**^**db**^**C**, was synthesised and isolated according to a previously reported procedure^13^. The guest molecules, **adm** and **adm**-*d*_16_, were purchased from FUJIFILM Wako Pure Chemical Corp. and CDN Isotopes, respectively.

### ^1^H NMR analyses of the hybrid nanocarbon assembly

Solution-phase ^1^H NMR spectra were recorded with a JEOL RESONANCE JNM-ECAII 600 spectrometer (600 MHz for ^1^H) equipped with an UltraCOOL probe. For the variable-temperature (VT) NMR analyses, a ROYAL probe was used. The assembly of **[3]C**^**db**^**C**⊃**adm** was confirmed by observations of upfield shifts of ^1^H resonances upon mixing a solution of **[3]C**^**db**^**C** (1.0 mM, 0.30 mL in CD_2_Cl_2_) and a solution of **adm** (1.0 mM, 0.30 mL in CD_2_Cl_2_) at 298 K (Fig. 2b and Supplementary Fig. 4). A reference spectrum of free-form **adm** was likewise recorded in CD_2_Cl_2_ at 298 K. The activation energy for the in-and-out exchange of **[3]C**^**db**^**C**⊃**adm** was estimated by VT NMR analyses: a 1:2 mixture of **[3]C**^**db**^**C** (0.33 mM) and **adm** (0.66 mM) in CD_2_Cl_2_ was analysed by ^1^H NMR spectroscopy in a temperature range of 183–298 K (Supplementary Fig. 1). Two sets of resonances of **[3]C**^**db**^**C**⊃**adm** and **adm** were observed at 183 K and coalesced at 203 K, which afforded a rate constant of dissociation (*k*_dis_) of 5.7 × 10^3^ s^–1^ and an activation energy (Δ*G*^‡^) of +8.2 kcal mol^–1^ via the coalescence method^24,34^. The 1:1 stoichiometry of the association of **[3]C**^**db**^**C** and **adm** was determined by Job plot analysis with ^1^H NMR spectra at 298 K. A series of solutions of **[3]C**^**db**^**C** and **adm** with variable molar ratios were prepared at a total concentration of 1.00 mM in CD_2_Cl_2_ (Supplementary Fig. 2). For the Job plot, methylene (CH_2_) resonances were adopted, as methine (CH) resonances were not easily located. Resonances of CH_2_ free-form **adm** appeared at 1.76 ppm and shifted upfield upon mixing **[3]C**^**db**^**C**, and by using the change in chemical shifts (Δ*δ*), the Job plot was obtained (Supplementary Fig. 3).

### ITC analysis

The association thermodynamics of **[3]C**^**db**^**C**⊃**adm** were investigated by performing ITC titrations on a Malvern MicroCal iTC200 instrument. A solution of **adm** (250 mM in CH_2_Cl_2_) was added to a solution of **[3]C**^**db**^**C** (0.499 mM in CH_2_Cl_2_) in a cell via a syringe for automated titration. The association parameters, *K*_a_, Δ*H*, and Δ*S*, were derived by using the ORIGIN software programme. The titration experiments were conducted in triplicate, and the averaged values and the standard deviation were reported. Isotherms are shown in Fig. 2c and Supplementary Fig. 5.

### Crystallographic analyses

A single crystal of **[3]C**^**db**^**C**⊃**adm** was grown from a 1:10 mixture of **[3]C**^**db**^**C** and **adm** in a solution of CH_2_Cl_2_/acetonitrile (*ca*. 1/1) at 298 K (*ca*. 2 mg/mL for the mixture). A single crystal of free-form **[3]C**^**db**^**C** was grown from nitrobenzene (*ca*. 1 mg/mL) via the gradual introduction of acetonitrile by vapour diffusion at 298 K. The single crystal was mounted on a thin polymer tip with cryoprotectant oil and frozen via flash cooling. Diffraction analyses with synchrotron X-ray sources were conducted under the following conditions: beamline = KEK Photon Factory BL17A, wavelength = 0.90000 Å, detector = Dectris EIGER X 16 M PAD, temperature = 95 K (**[3]C**^**db**^**C**⊃**adm**); beamline = SPring-8 BL38B1, wavelength = 0.80000 Å, detector = Dectris PILATUS3 6 M PAD, temperature = 100 K (free-form **[3]C**^**db**^**C**). The collected diffraction data were processed with the XDS software programme^35^. The structure was solved by direct methods with SHELXT^36^ and refined by full-matrix least squares on *F*^2^ using the SHELXL-2014/7 programme suite^37^ running with Yadokari-XG 2009^38^. In the refinements, **adm**, disordered alkyl chains, and solvent molecules were restrained by SIMU, ISOR, DFIX, and DANG. The nonhydrogen atoms were analysed anisotropically, and hydrogen atoms were located at the calculated positions and refined with a riding model. The crystal/refinement data are listed in Supplementary Tables 1 and 2. Crystal structures are shown in Fig. 3 and Supplementary Fig. 7-11. The Hirshfeld analyses were performed using the CrystalExplorer software programme^39^.

### Theoretical calculations

Theoretical DFT calculations of **[3]C**^**db**^**C**⊃**adm** with a methyl-substituted cylinder as the model were performed with the LC-BLYP functional^40^ and the 6-311 G(d) basis set^41^ via counterpoise BSSE corrections in Gaussian 16^42^. The AIM analyses were performed with Multiwfn^43^. The global minimum structure at (A,B) = (0,0) was obtained by geometry optimisations using a crystal structure adopted as the initial geometry. The energy profile was obtained by performing single-point energy calculations of 324 (18 × 18) orientations in total by rotating **adm** 20° along axis A and axis B (Fig. 4). The number of CH–π hydrogen bonds was manually counted for 5184 hydrogen atoms to visualise the hydrogen-bonding sites among the 324 orientations (Supplementary Fig. 12).

### Solid-state ^2^H NMR analyses

Solid-state ^2^H NMR spectra for the temperature range of 200–390 K were obtained on a JEOL RESONANCE JNM-ECAII 600 spectrometer (92.1 MHz for ^2^H) equipped with a 3.2-mm HXMAS probe. The spectra were recorded in the temperature range of 420–560 K using a JEOL RESONANCE JNM-ECA 500 spectrometer (76.8 MHz for ^2^H) equipped with a homemade probe provided by the NIMS microstructural characterisation platform. A 1:1 mixture of **[3]C**^**db**^**C** (45.08 mg, 30.3 *µ*mol) and **adm**-*d*_16_ (4.62 mg, 30.3 *µ*mol) was prepared in a solution of CH_2_Cl_2_/acetonitrile (4/1) and dried in vacuo for 1 h after removal of the solvent. The powder specimen was loaded in a 3.2-mm ZrO_2_ NMR tube (200–390 K) or sealed in a glass tube (420–560 K), and spectra were recorded under MAS and static conditions (Fig. 5). A simulated ^2^H spectrum of motionless, static **adm**-*d*_16_ was obtained by using NMR WEBLAB (Fig. 5)^44^. The spin-lattice relaxation time *T*_1_ was then measured by a saturation-recovery method in a temperature range of 200–560 K under static conditions without MAS (Supplementary Fig. 13). The specimen after the *T*_1_ measurements was examined by recording the ^2^H NMR spectrum, which afforded an identical spectrum before the *T*_1_ measurement. The rotational correlation time *τ* was calculated by using

1 $$\frac{1}{{T}_{1}}=\frac{3}{40}\left(1+\frac{{\eta }^{2}}{3}\right){\left(2{{\pi }}\cdot \frac{{e}^{2}{{{{{\rm{Qq}}}}}}}{h}\right)}^{2}\left(\frac{\tau }{1+{\omega }^{2}{\tau }^{2}}+\frac{4{{\tau }}}{1+4{\omega }^{2}{\tau }^{2}}\right)$$

where *e*^2^*Qq*/*h* is a quadrupole-coupling constant (174 kHz), *η* is the asymmetric parameter of the electron-field-gradient tensor (0), and *ω* is the Larmor frequency (92.1 or 76.8 MHz)^45,46^. The temperature dependence of the rotational frequency (*k*_rot_ = *τ*^−1^) was analysed by the Eyring plot to obtain the activation parameters (Fig. 5c). For the *χ*-test, the moment of inertia of **adm**-*d*_16_ rotations was calculated as 6.21 × 10^–45^ kg m^2^ to derive theoretical *τ*_FR_ values for the free rotations^27^. Supplementary Table 4 summarises the *T*_1_, *τ*, *k*_rot_, and *χ* values.

## Supplementary information

Supplementary Information

Description of Additional Supplementary Files

Supplementary Movie 1

Supplementary Movie 2

## Data Availability

Supplementary spectra and computational data are provided in the Supplementary Information. Crystallographic data of **[3]C**^**db**^**C**⊃**adm** and guest-free **[3]C**^**db**^**C** have been deposited at the Cambridge Crystallographic Data Centre with deposition number CCDC 2072466 and 1874315. These data can be obtained free of charge from the Cambridge Crystallographic Data Centre via www.ccdc.cam.ac.uk/structures/.
