# Supplementary material for: A hybrid molecular peapod of sp2- and sp3-nanocarbons enabling ultrafast terahertz rotations
Source: Nat Commun. 2021 Aug 25;12:5062. doi: 10.1038/s41467-021-25358-0 (PMC8387501; doi:10.1038/s41467-021-25358-0)
Supplement: Supplementary file 1 — Supplementary Information [file 41467_2021_25358_MOESM1_ESM.pdf]

Supplementary Information

A hybrid molecular peapod of sp^2- and sp^3-nanocarbons enabling ultrafast terahertz rotations

Taisuke Matsuno[1*], Seiya Terasaki[1], Kanako Kogashi[2], Ryosuke Katsuno[1] and Hiroyuki Isobe[1*]

[1]Department of Chemistry, The University of Tokyo, Hongo 7-3-1, Bunkyo-ku, Tokyo 113-0033, Japan

[2]Department of Chemistry, Tohoku University, Aoba-ku, Sendai 980-8578, Japan

Table of Contents

Supplementary Figures..2

Supplementary Tables..12

Supplementary Figures

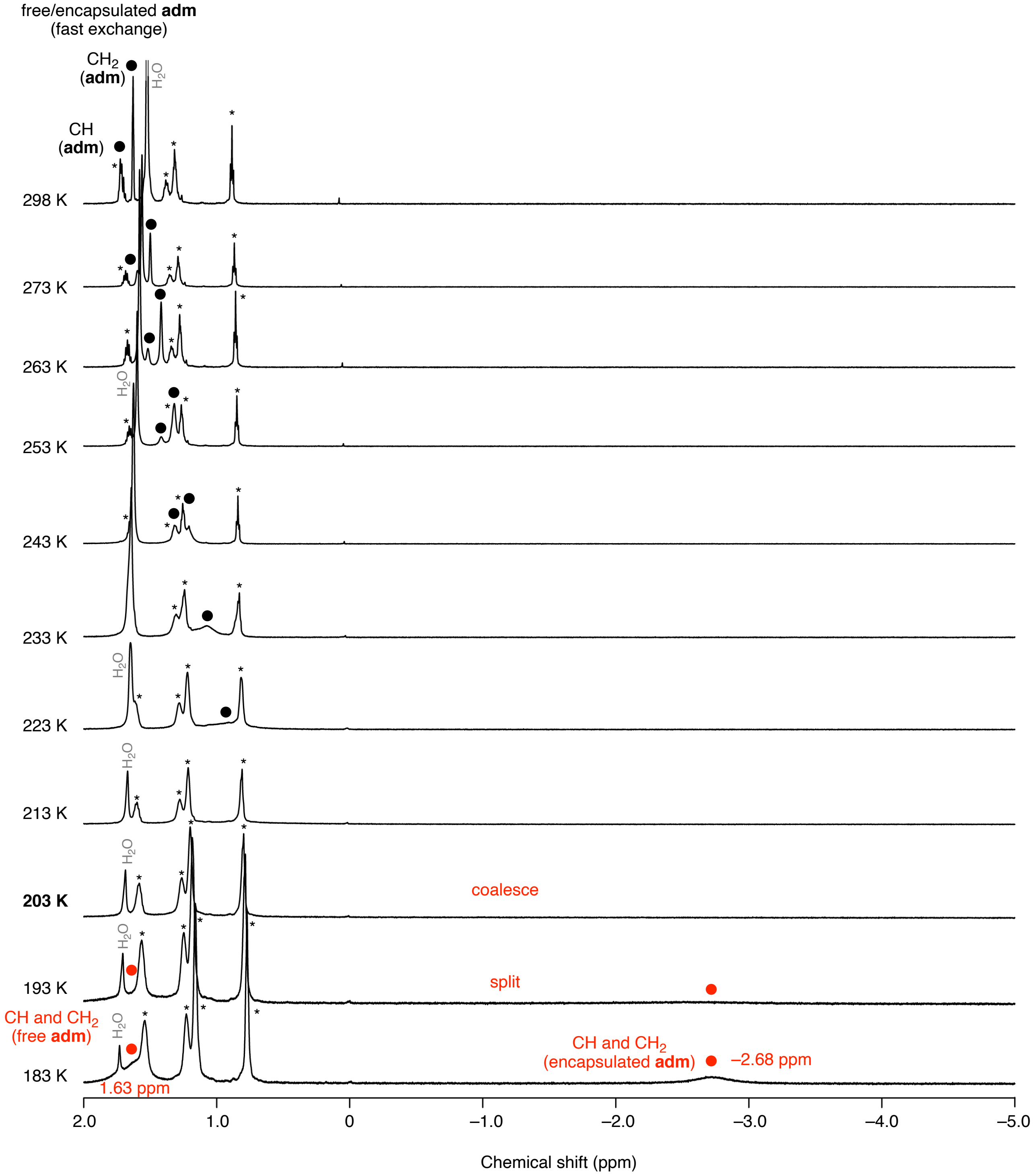

Supplementary Fig. 1 | VT ^{1}H NMR spectra of 1:2 mixture of [3]C^{db}C and adm in CD_2Cl_2. The resonances of **adm** are labeled with dots that are coloured in black for time-averaged resonances under rapid in-and-out exchange and in red for separate two sets of resonances under retarded in-and-out exchange. Resonances of hexyl chains of **[3]C^{db}C** are labeled with asterisks.

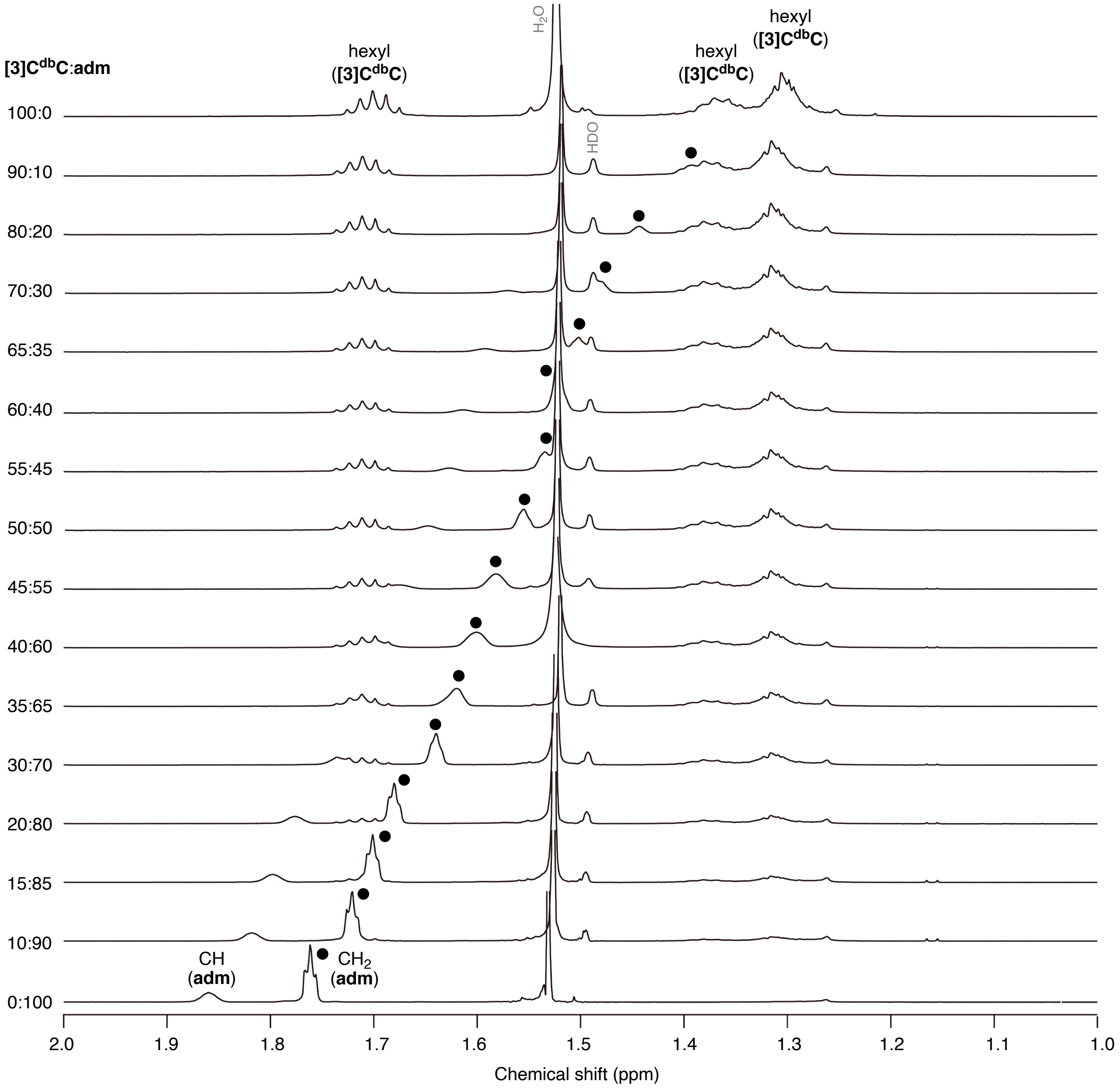

Supplementary Fig. 2 | ^{1}H NMR spectra of [3]C^{db}C⊃adm for the Job plot analysis. Spectra were recorded in CD_2Cl_2 at a total concentration of 1.0 mM (298 K). Methylene (CH_2) resonances of **adm** were used for the Job plot analysis (black dots).

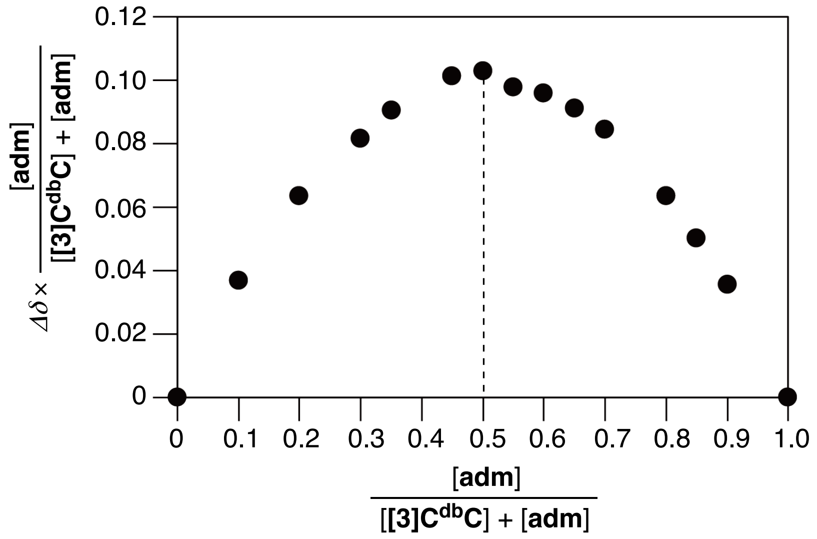

Supplementary Fig. 3 | Job plot of from ^{1}H NMR analyses. The peak at 0.5 confirmed the formation of 1:1 complex of **[3]C^{db}C⊃adm**. See Supplementary Fig. 2 for the spectra.

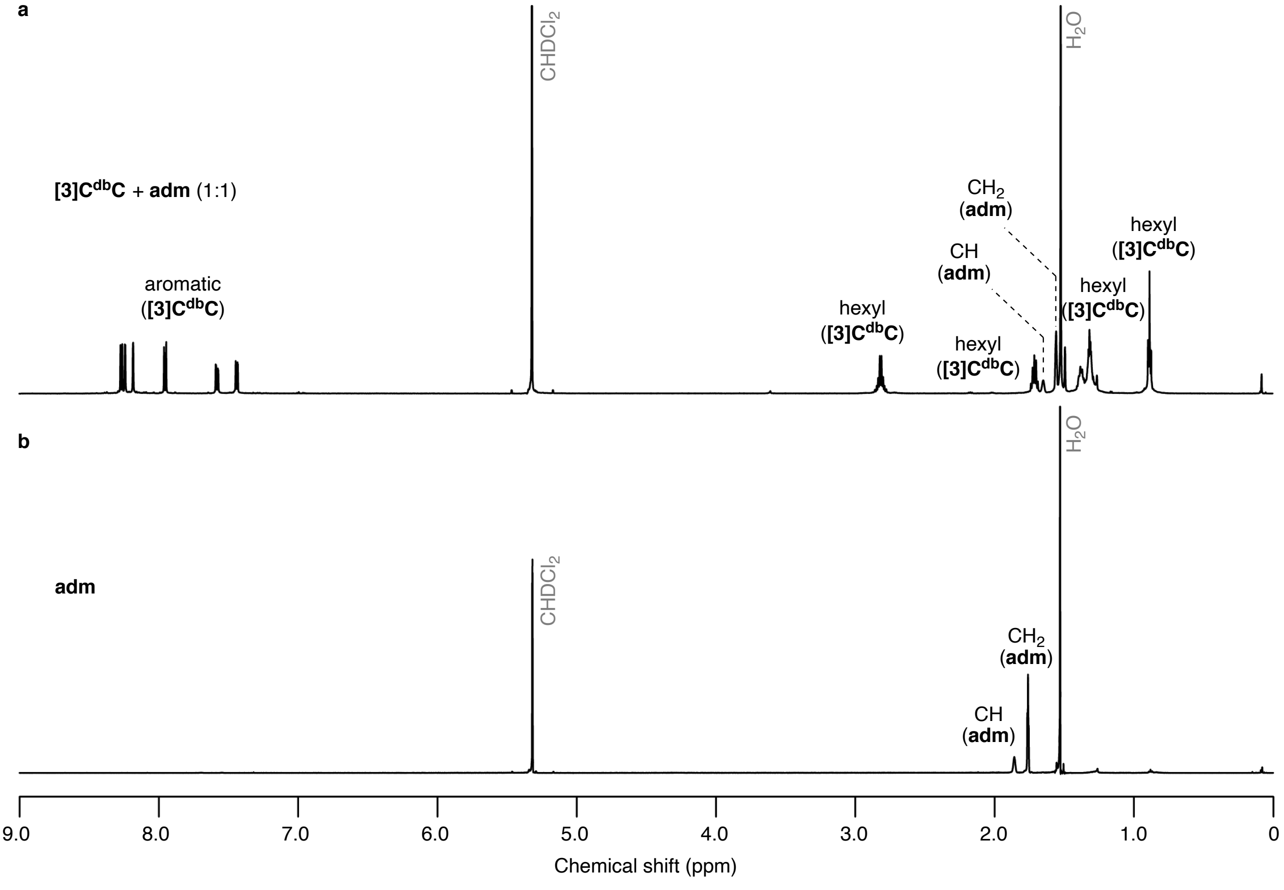

Supplementary Fig. 4 | ^{1}H NMR spectra in CD_2Cl_2 at 298 K (whole region). See Fig. 2 for close-up views. **a**, A spectrum of **[3]C^{db}C⊃adm**. **b**, A reference spectrum of free-form **adm.**

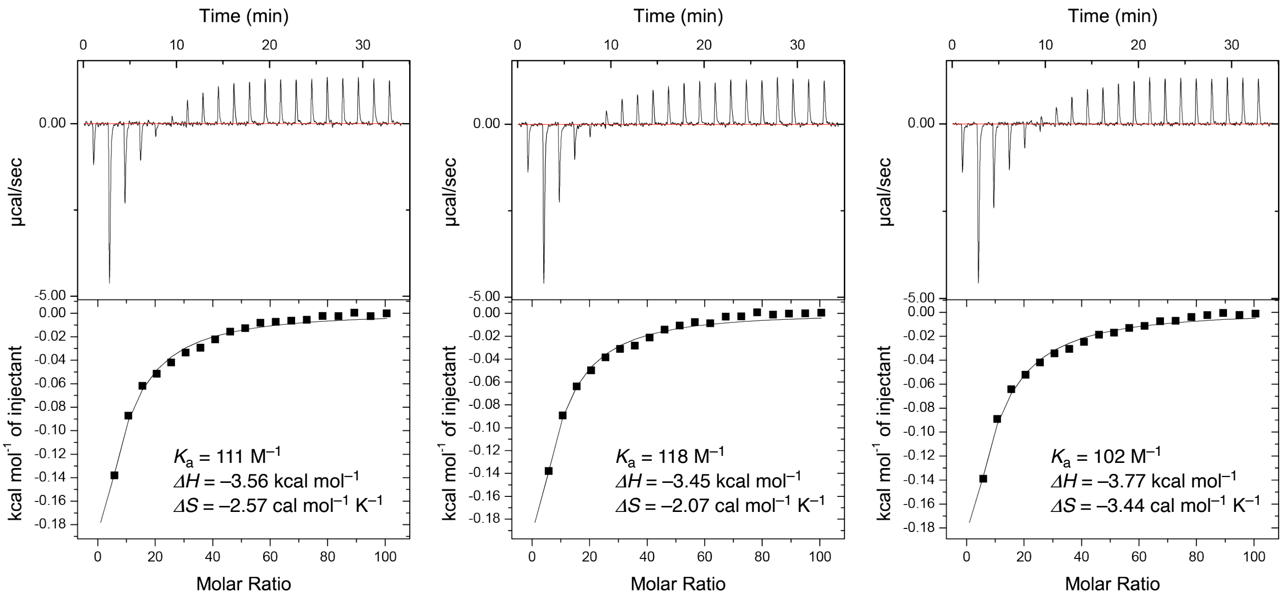

Supplementary Fig. 5 | Raw ITC data of triplicate titrations. Titrations were performed in CH_2Cl_2 at 298 K, and the association parameters of each titration are shown.

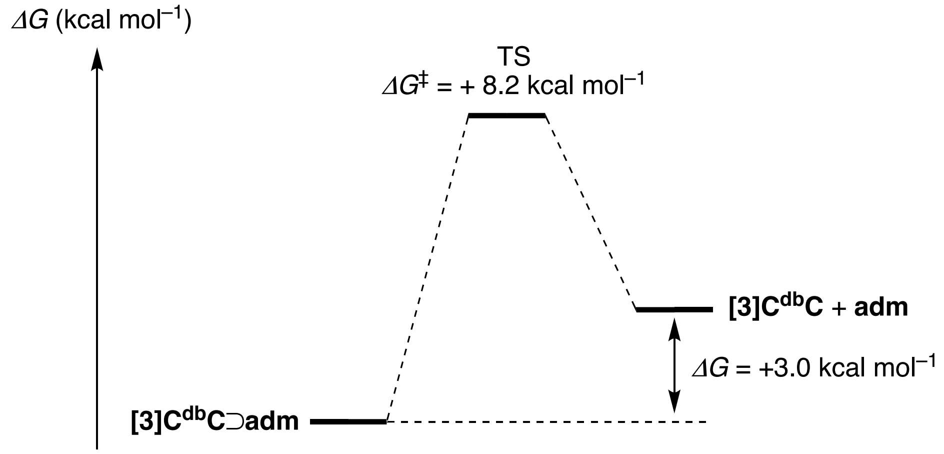

Supplementary Fig. 6 | Energetics for assembly of [3]C^{db}C⊃adm. The thermodynamic parameter of ΔG was determined by ITC (Fig. 2c and Supplementary Fig. 5), and the kinetic barrier of $\Delta G^{\ddagger}$ was determined by NMR analyses (Supplementary Fig. 1).

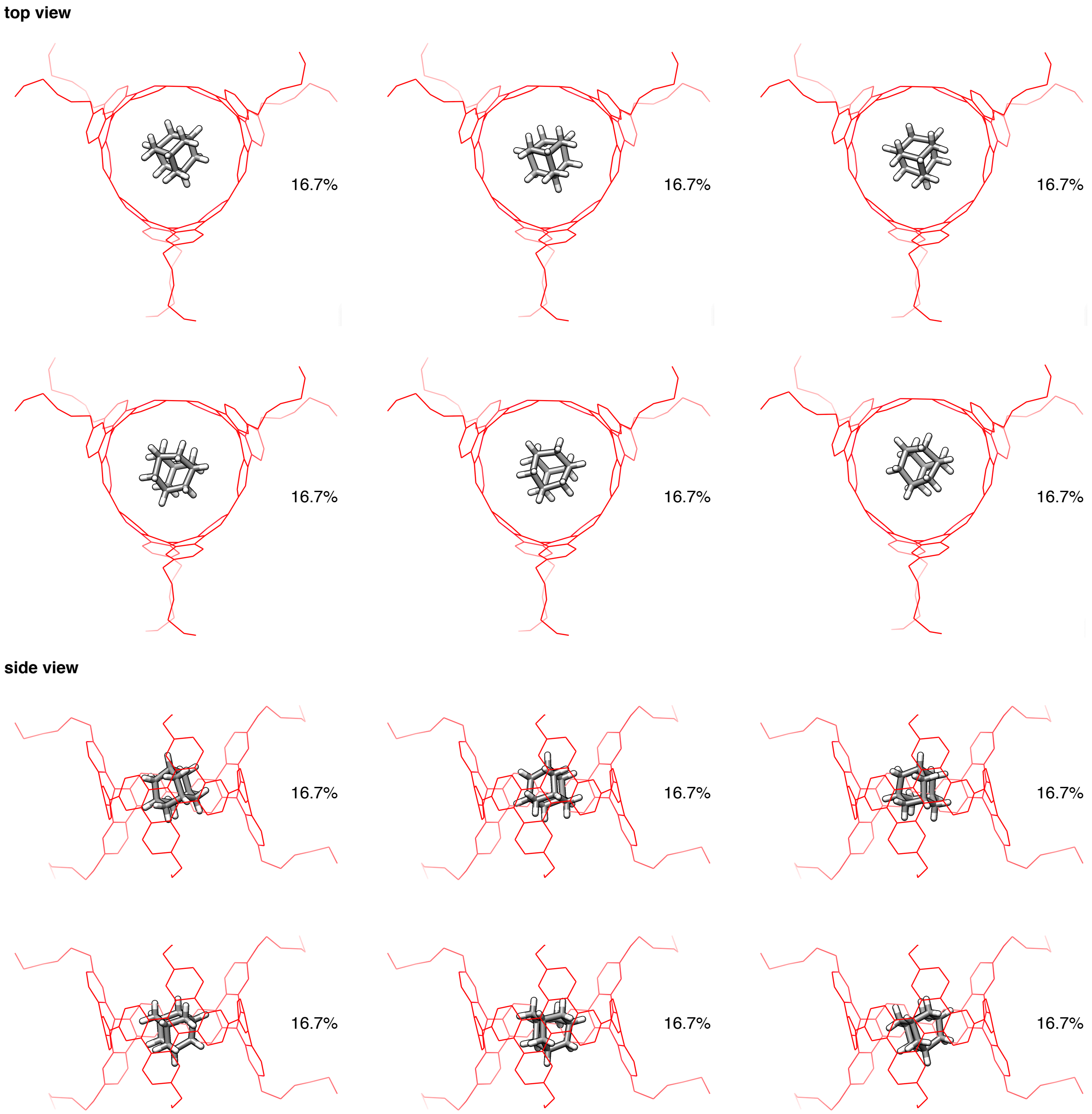

Supplementary Fig. 7 | Orientations of adm guests found as disordered structures in [3]C^{db}C$\supset$adm. Six orientations are located in **[3]C^{db}C**, and the occupancy values are shown.

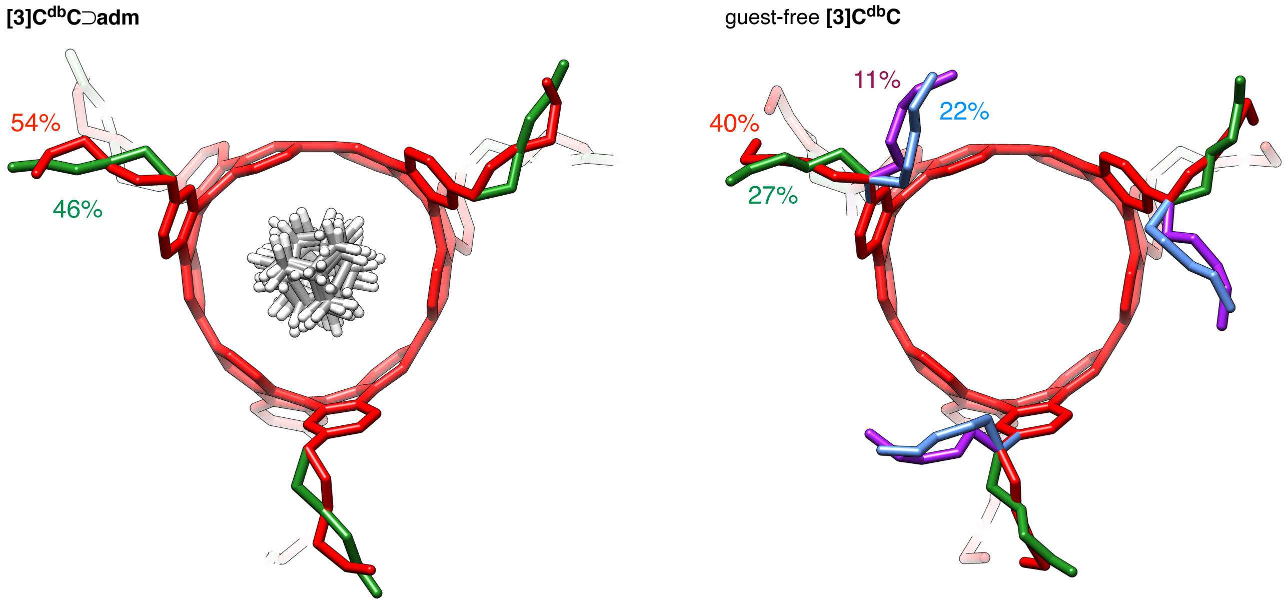

Supplementary Fig. 8 | Conformations of hexyl side chains found as disordered structures. Minor conformers are shown in green, blue and purple, and the occupancy values are shown.

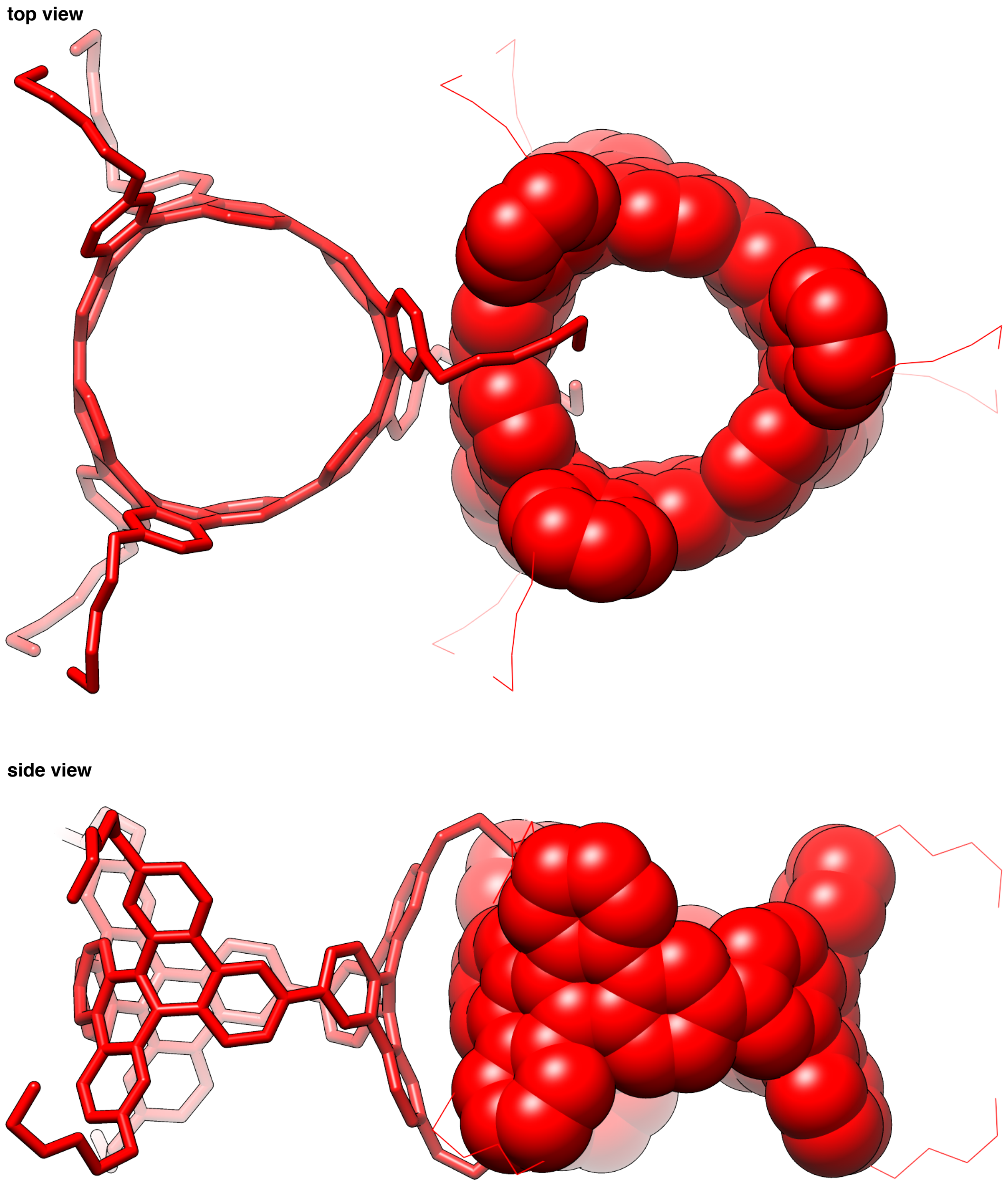

Supplementary Fig. 9 | Structural relationships between neighbouring [3]C^{db}C molecules in the crystal. Two hexyl chains of one molecule pinch the wall of another neighbouring molecule. See also Fig. 3 and Supplementary Fig. 10 for the resultant packings.

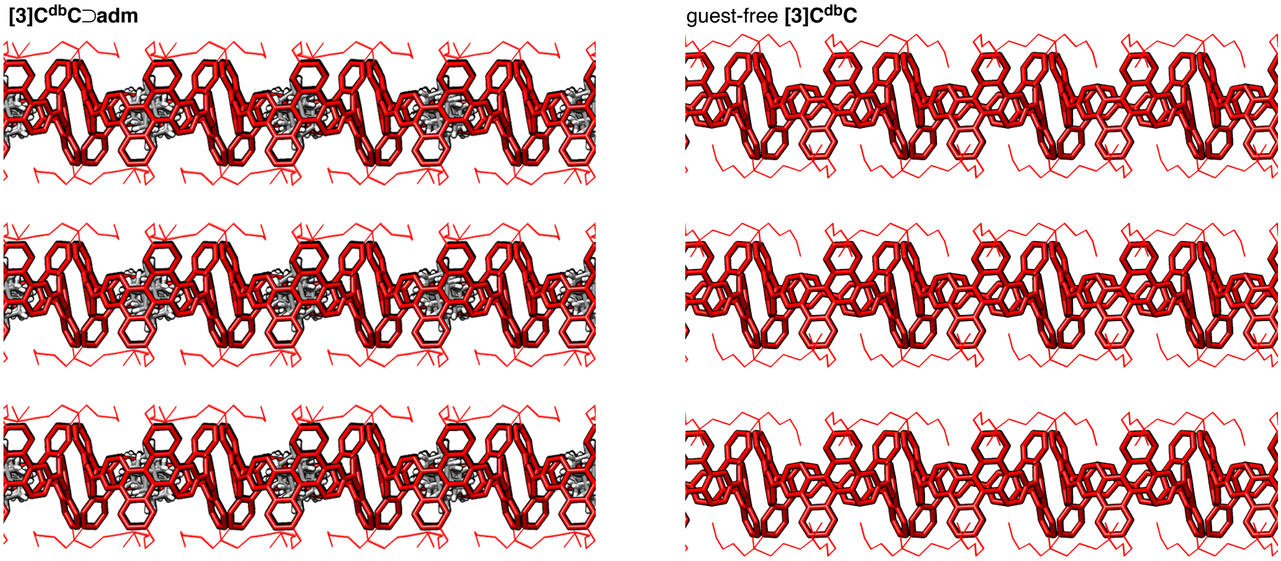

Supplementary Fig. 10 | Packing structures. Two-dimensional layers of **[3]C^{db}C** molecules are stacked to form a single crystal in the presence and the absence of the guest molecules. See also Fig. 3 for the two-dimensional layer.

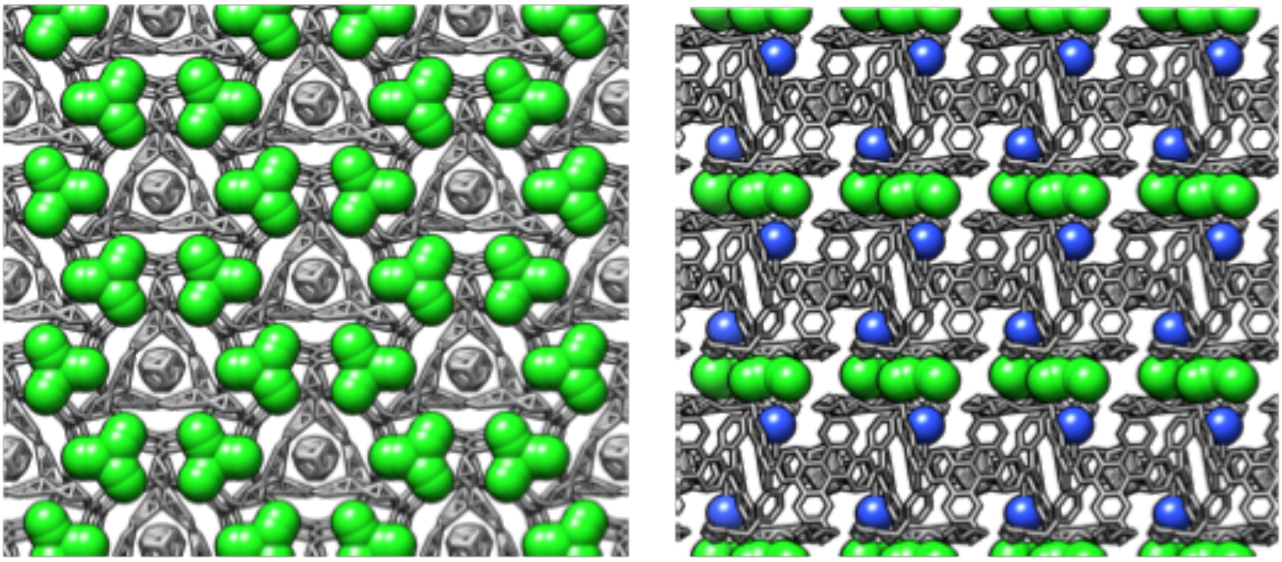

Supplementary Fig. 11 | Solvent molecules in the crystal. Solvent molecules (dichloromethane and acetonitrile) were included in a space surrounded by host-guest complexes and had no direct contact with guest molecules. Chlorine atoms of dichloromethane and nitrogen atoms of acetonitrile are shown as ball models (green = Cl, blue = N).

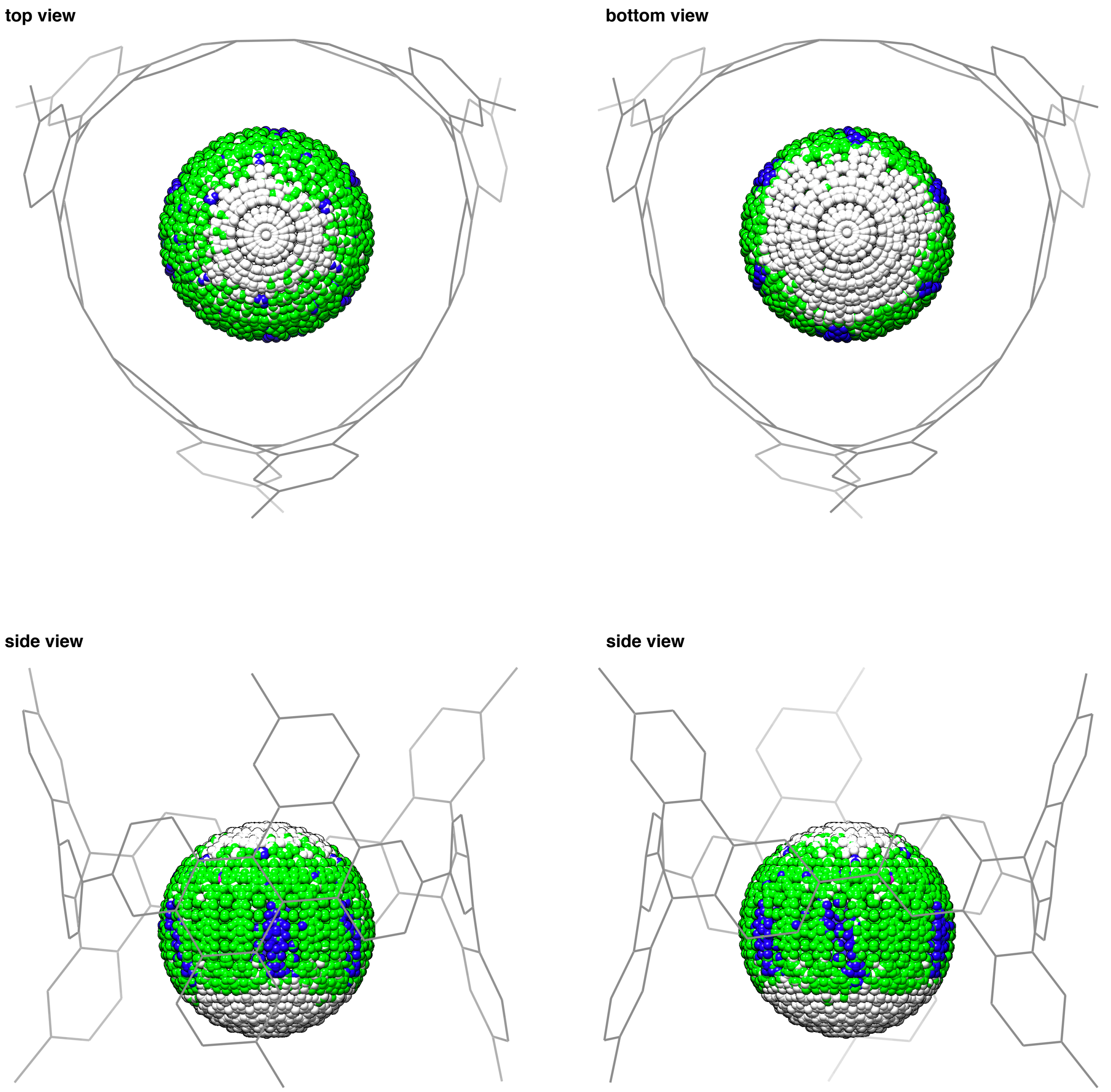

Supplementary Fig. 12 | CH-π hydrogen bonding continuum circumfused by the carbonaceous cylinder. The 5184 hydrogen atoms of **adm** for 324 orientations within **[3]C$^{\mathbf{db}}$C** (Fig. 4b) were analysed by AIM calculations for the hydrogen bonds, and the number of hydrogen bonds were counted. The number of hydrogen bonds were shown in colours (3 bonds = magenta, 2 bonds = blue, 1 bond = green, 0 bond = white).

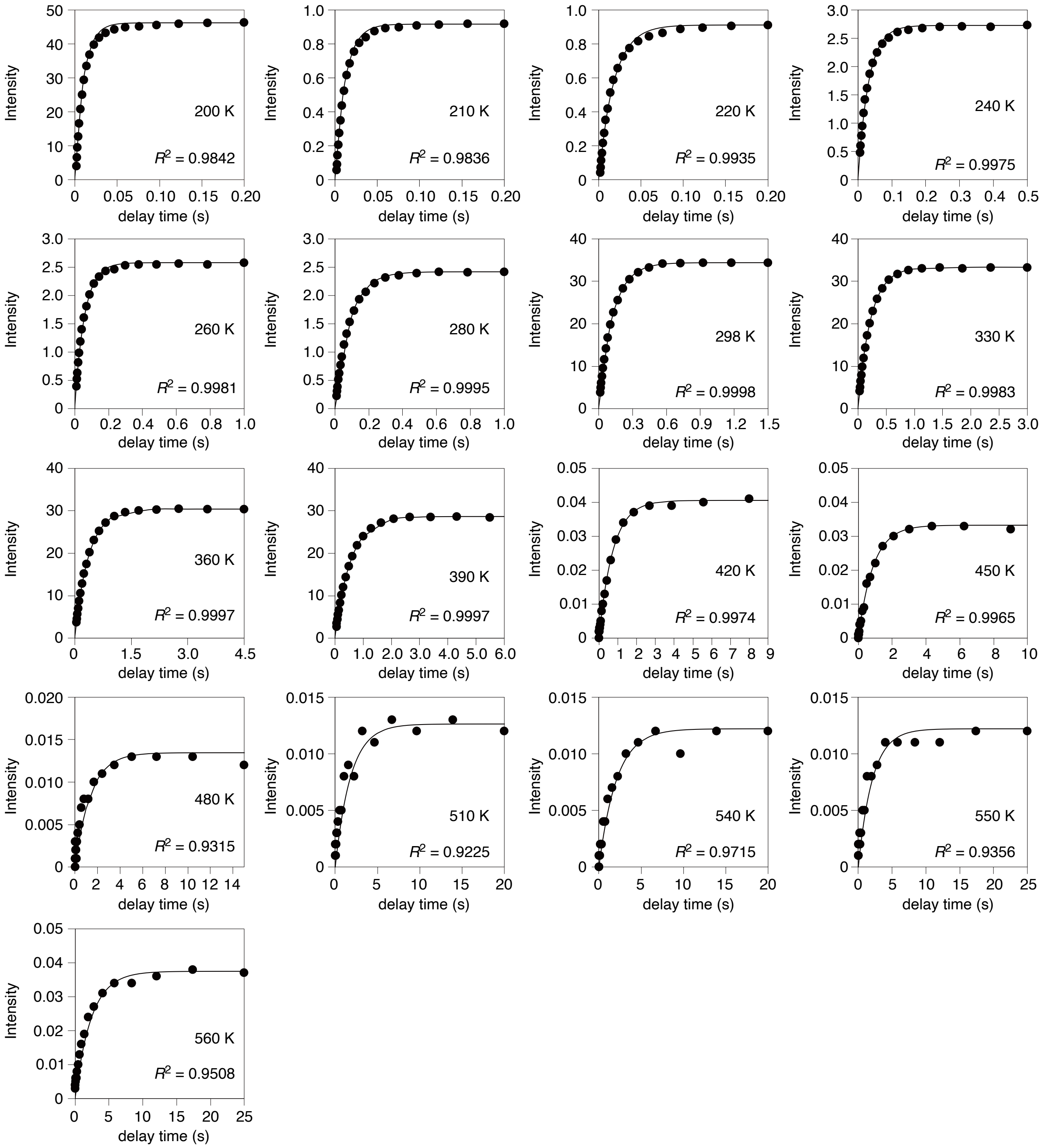

Supplementary Fig. 13 | Raw saturation-recovery data for T_1 values. Temperatures were varied in a range of 200-560 K. The coefficients of determination in R^2 are shown.

Supplementary Tables

Supplementary Table 1 | Crystal data and structure refinement for [3]C^{db}C⊃adm.

CCDC No.	2072466
Empirical formula	$C_{130} H_{140} Cl_4 N_2$
Formula weight	1872.23
Temperature	95(2) K
Wavelength	0.90000 Å
Crystal system	Trigonal
Space group	*P*321
Unit cell dimensions	a = 14.120(2) Å α = 90°.
	b = 14.120(2) Å β = 90°.
	c = 15.130(3) Å γ = 120°.
Volume	2612.4(9) Å^3
Z	1
Density (calculated)	1.190 Mg/m^3
Absorption coefficient	0.305 mm^{-1}
F(000)	1002
Crystal size	0.100 × 0.100 × 0.050 mm^3
Theta range for data collection	2.109 to 32.855°.
Index ranges	–17<=*h*<=17, –15<=*k*<=15, –18<=*l*<=18
Reflections collected	28914
Independent reflections	3198 [*R*(int) = 0.0452]
Completeness to theta = 32.684°	99.6 %
Absorption correction	Semi-empirical from equivalents
Max. and min. transmission	1.000 and 0.693
Refinement method	Full-matrix least-squares on F^2
Data / restraints / parameters	3198 / 288 / 347
Goodness-of-fit on F^2	1.385
Final *R* indices [*I*>2sigma(*I*)]	R_1 = 0.0878, wR_2 = 0.2584
R indices (all data)	R_1 = 0.0879, wR_2 = 0.2587
Absolute structure parameter	0.10(4)
Extinction coefficient	0.21(3)
Largest diff. peak and hole	0.372 and –0.603 e.Å^{-3}

Supplementary Table 2 | Crystal data and structure refinement for guest-free [3]C^{db}C.

CCDC No.	1874315
Empirical formula	$C_{126}H_{132}N_6$
Formula weight	1730.37
Temperature	100(2) K
Wavelength	0.80000 Å
Crystal system	Trigonal
Space group	*P*321
Unit cell dimensions	a = 14.070(2) Å α = 90°
	b = 14.070(2) Å β = 90°
	c = 15.560(3) Å γ = 120°
Volume	2667.6(9) Å^3
Z	1
Density (calculated)	1.077 Mg/m^3
Absorption coefficient	0.078 mm^{-1}
F(000)	930
Crystal size	0.200 × 0.100 × 0.100 mm^3
Theta range for data collection	2.390 to 29.962°
Index ranges	$-17 <= h <= 17, -17 <= k <= 17, -18 <= l <= 18$
Reflections collected	31268
Independent reflections	3598 [*R*(int) = 0.0312]
Completeness to theta = 28.685°	99.7 %
Absorption correction	Semi-empirical from equivalents
Max. and min. transmission	1.000 and 0.852
Refinement method	Full-matrix least-squares on F^2
Data / restraints / parameters	3598 / 266 / 376
Goodness-of-fit on F^2	1.053
Final *R* indices [*I*>2sigma(*I*)]	R_1 = 0.0676, wR_2 = 0.1817
R indices (all data)	R_1 = 0.0703, wR_2 = 0.1892
Absolute structure parameter	–1.3(10)
Extinction coefficient	n/a
Largest diff. peak and hole	0.216 and –0.285 e•Å^{-3}

Supplementary Table 3 | Cartesian coordinates of methyl-substituted [3]C^{db}C⊃adm.

SCF Done: E(RLC-B+HF-LYP) = -3613.83517924 A.U. after 7 cycles

Center Number	Atomic Number	Atomic Type	Coordinates (Angstroms) X	Y	Z
1	6	0	3.119619	-3.923483	0.057443
2	6	0	2.445867	-4.499599	1.110143
3	1	0	2.797631	-4.352127	2.121250
4	6	0	1.209082	-5.117224	0.953441
5	6	0	0.618323	-5.187307	-0.308611
6	6	0	1.402139	-4.788858	-1.396396
7	1	0	0.999648	-4.828114	-2.397733
8	6	0	2.609768	-4.179900	-1.218079
9	1	0	3.108842	-3.795157	-2.096695
10	6	0	-1.540480	-5.360558	-1.674512
11	6	0	-2.794586	-4.766517	-1.778290
12	6	0	-3.497836	-4.880424	-2.976392
13	1	0	-4.484437	-4.439898	-3.059041
14	6	0	-2.985351	-5.580270	-4.029672
15	1	0	-3.558118	-5.670835	-4.946958
16	6	0	-1.747466	-6.214102	-3.929758
17	6	0	-1.048847	-6.093360	-2.758592
18	1	0	-0.109876	-6.622974	-2.650931
19	6	0	-0.830069	-5.298583	-0.408112
20	6	0	-4.512111	-2.142592	0.271283
21	6	0	-4.163666	-2.957657	-0.781216
22	1	0	-4.430021	-2.676915	-1.790320
23	6	0	-3.315038	-4.051030	-0.629107
24	6	0	-2.806843	-4.369346	0.630079
25	6	0	-3.314047	-3.652995	1.719608
26	1	0	-2.951531	-3.857107	2.716261
27	6	0	-4.134846	-2.578243	1.544873
28	1	0	-4.397004	-2.002856	2.421852
29	6	0	-0.962397	-5.497124	1.995461

30	6	0	0.423845	-5.526575	2.101774
31	6	0	1.000801	-5.944751	3.299585
32	1	0	2.080303	-5.991904	3.382963
33	6	0	0.227357	-6.343687	4.350740
34	1	0	0.697421	-6.683264	5.268044
35	6	0	-1.163236	-6.355761	4.248786
36	6	0	-1.732323	-5.932820	3.077516
37	1	0	-2.809206	-5.982725	2.968660
38	6	0	-1.565537	-5.121886	0.727655
39	6	0	4.111306	-2.835586	0.272666
40	6	0	4.643307	-2.126801	-0.780002
41	1	0	4.533367	-2.498109	-1.789002
42	6	0	5.166249	-0.845290	-0.628237
43	6	0	5.187859	-0.245634	0.630766
44	6	0	4.820698	-1.042580	1.720515
45	1	0	4.816181	-0.626205	2.717014
46	6	0	4.299920	-2.290649	1.546087
47	1	0	3.932449	-2.805035	2.423164
48	6	0	5.242638	1.916067	1.995453
49	6	0	4.575306	3.131519	2.101380
50	6	0	4.649369	3.840724	3.298883
51	1	0	4.150704	4.799338	3.381967
52	6	0	5.381587	3.370546	4.350131
53	1	0	5.440916	3.947765	5.267203
54	6	0	6.086933	2.172026	4.248600
55	6	0	6.004880	1.467297	3.077599
56	1	0	6.586236	0.559401	2.969043
57	6	0	5.219045	1.205663	0.727903
58	6	0	-4.958666	-0.739915	0.055919
59	6	0	-5.121875	0.131357	1.108669
60	1	0	-5.170800	-0.247252	2.119650
61	6	0	-5.038391	1.511294	0.952350
62	6	0	-4.802902	2.058284	-0.309425
63	6	0	-4.848716	1.180514	-1.397449
64	1	0	-4.680709	1.549018	-2.398559

65	6	0	-4.924800	-0.169868	-1.219455
66	1	0	-4.840050	-0.794270	-2.098110
67	6	0	-3.872721	4.014986	-1.674152
68	6	0	-2.731198	4.804173	-1.777190
69	6	0	-2.477969	5.470817	-2.974864
70	1	0	-1.603189	6.105079	-3.056956
71	6	0	-3.339995	5.377394	-4.028436
72	1	0	-3.131828	5.919203	-4.945382
73	6	0	-4.507777	4.622044	-3.929293
74	6	0	-4.752791	3.956022	-2.758546
75	1	0	-5.680873	3.407437	-2.651434
76	6	0	-4.174778	3.368177	-0.408187
77	6	0	1.837863	4.662529	0.057214
78	6	0	2.674393	4.368436	1.109738
79	1	0	2.371288	4.600269	2.120795
80	6	0	3.827813	3.606371	0.953032
81	6	0	4.183473	3.129130	-0.308887
82	6	0	3.445807	3.607571	-1.396679
83	1	0	3.680695	3.277907	-2.397870
84	6	0	2.314269	4.348367	-1.218310
85	1	0	1.730751	4.586932	-2.096752
86	6	0	5.412893	1.345924	-1.674341
87	6	0	5.525665	-0.037193	-1.777691
88	6	0	5.975799	-0.589577	-2.975701
89	1	0	6.087710	-1.664274	-3.058051
90	6	0	6.325299	0.203910	-4.029297
91	1	0	6.690002	-0.247059	-4.946520
92	6	0	6.254990	1.592890	-3.929844
93	6	0	5.801250	2.137831	-2.758762
94	1	0	5.790078	3.215851	-2.651456
95	6	0	5.004188	1.930529	-0.408075
96	6	0	0.399818	4.976816	0.272799
97	6	0	-0.480179	5.084257	-0.779624
98	1	0	-0.103724	5.176046	-1.788545
99	6	0	-1.851446	4.896156	-0.627827

100	6	0	-2.381388	4.614069	0.631041
101	6	0	-1.507365	4.693015	1.720689
102	1	0	-1.865560	4.479771	2.717001
103	6	0	-0.166126	4.865978	1.546185
104	1	0	0.463300	4.803316	2.422988
105	6	0	-4.281065	3.580959	1.995501
106	6	0	-5.000463	2.395571	2.101057
107	6	0	-5.651561	2.104845	3.298567
108	1	0	-6.232723	1.193859	3.381416
109	6	0	-5.609960	2.973622	4.350151
110	1	0	-6.139430	2.736223	5.267226
111	6	0	-4.924215	4.183502	4.248991
112	6	0	-4.272955	4.465023	3.078000
113	1	0	-3.776870	5.422219	2.969783
114	6	0	-3.654101	3.915953	0.727993
115	6	0	6.926151	1.695381	5.391713
116	1	0	7.690609	2.429143	5.652848
117	1	0	6.319107	1.530405	6.283775
118	1	0	7.431423	0.759397	5.156224
119	6	0	6.690900	2.450876	-5.075208
120	1	0	7.730981	2.255141	-5.341461
121	1	0	6.088051	2.256291	-5.964142
122	1	0	6.602447	3.510837	-4.839651
123	6	0	-5.468897	4.571266	-5.074582
124	1	0	-5.819303	5.570054	-5.340304
125	1	0	-4.999121	4.146846	-5.963779
126	1	0	-6.342689	3.964699	-4.839227
127	6	0	-4.930456	5.148136	5.392504
128	1	0	-5.947950	5.444022	5.653574
129	1	0	-4.484591	4.704180	6.284466
130	1	0	-4.371732	6.053354	5.157493
131	6	0	-1.995778	-6.844264	5.391774
132	1	0	-1.742534	-7.873180	5.652925
133	1	0	-1.835275	-6.236069	6.283865
134	1	0	-3.058974	-6.813906	5.156149

135	6	0	-1.222302	-7.021084	-5.074749
136	1	0	-1.912325	-7.823400	-5.341439
137	1	0	-1.088308	-6.401838	-5.963614
138	1	0	-0.260637	-7.475220	-4.838553
139	6	0	-1.132132	0.884338	-0.170149
140	1	0	-2.092944	0.519666	0.206432
141	1	0	-1.012133	1.906532	0.202609
142	6	0	-1.136107	0.882465	-1.694602
143	1	0	-1.949856	1.516340	-2.058173
144	6	0	0.002612	0.003222	0.341012
145	1	0	0.003238	0.005094	1.434692
146	6	0	-0.194410	-1.421104	-0.166800
147	1	0	-1.138662	-1.827514	0.209292
148	1	0	0.602631	-2.070355	0.208970
149	6	0	-1.328032	-0.543253	-2.197589
150	1	0	-1.353687	-0.555724	-3.292388
151	1	0	-2.286874	-0.936233	-1.849674
152	6	0	-0.194655	-1.426780	-1.691246
153	1	0	-0.337507	-2.449191	-2.052376
154	6	0	1.134898	-0.881236	-2.198368
155	1	0	1.955402	-1.514383	-1.850897
156	1	0	1.156125	-0.899486	-3.293182
157	6	0	0.196062	1.420595	-2.201789
158	1	0	0.334889	2.448539	-1.856940
159	1	0	0.198708	1.445696	-3.296668
160	6	0	1.333416	0.543625	-0.171003
161	1	0	1.498180	1.559183	0.202441
162	1	0	2.158212	-0.070446	0.204291
163	6	0	1.334564	0.543096	-1.695454
164	1	0	2.290527	0.929819	-2.059842

Supplementary Table 4 | Key features of solid-state rotations of [3]C^{db}C⊃adm.

Temperature (K)	T_1 (s)	τ (ps)	k_{rot} (GHz)	χ
200	0.0103 ± 0.0005	217 ± 11	4.61 ± 0.24	241 ± 13
210	0.0122 ± 0.0007	184 ± 10	5.44 ± 0.31	210 ± 12
220	0.0182 ± 0.0007	123 ± 5	8.13 ± 0.31	143 ± 5
240	0.0318 ± 0.0007	70.2 ± 1.4	14.3 ± 0.3	85.4 ± 1.8
260	0.0580 ± 0.0010	38.5 ± 0.7	26.0 ± 0.4	48.7 ± 0.8
280	0.0890 ± 0.0009	25.1 ± 0.3	39.9 ± 0.4	33.0 ± 0.3
298	0.125 ± 0.001	17.9 ± 0.1	55.9 ± 0.3	24.3 ± 0.1
330	0.226 ± 0.004	9.87 ± 0.18	101 ± 2	14.1 ± 0.3
360	0.368 ± 0.003	6.07 ± 0.05	164 ± 1	9.04 ± 0.07
390	0.557 ± 0.004	4.00± 0.03	250 ± 2	6.21 ± 0.05
420	0.741 ± 0.024	3.02± 0.10	332 ± 11	4.86± 0.16
450	0.869 ± 0.033	2.57 ± 0.10	390 ± 15	4.29 ± 0.16
480	1.37 ± 0.21	1.66 ± 0.25	600 ± 92	2.87 ± 0.44
510	1.82 ± 0.30	1.25 ± 0.21	798 ± 136	2.23 ± 0.38
540	2.09 ± 0.22	1.09 ± 0.11	919 ± 93	1.99 ± 0.20
550	2.08 ± 0.29	1.09 ± 0.2	915 ± 130	2.01 ± 0.29
560	2.40 ± 0.30	0.948 ± 0.118	1060 ± 130	1.76 ± 0.21
