# Supplementary material for: A hybrid molecular peapod of sp2- and sp3-nanocarbons enabling ultrafast terahertz rotations
Source: Nat Commun. 2021 Aug 25;12:5062. doi: 10.1038/s41467-021-25358-0 (PMC8387501; doi:10.1038/s41467-021-25358-0)
Supplement: Supplementary file 2 — Description of Additional Supplementary Files [file 41467_2021_25358_MOESM2_ESM.docx]

**Supplementary Movie 1.** Rotation around axis A

**Supplementary Movie 2.** Rotation around axis B
