# Supplementary figures and images for: A hybrid molecular peapod of sp2- and sp3-nanocarbons enabling ultrafast terahertz rotations
Source: Nat Commun. 2021 Aug 25;12:5062. doi: 10.1038/s41467-021-25358-0 (PMC8387501; doi:10.1038/s41467-021-25358-0)

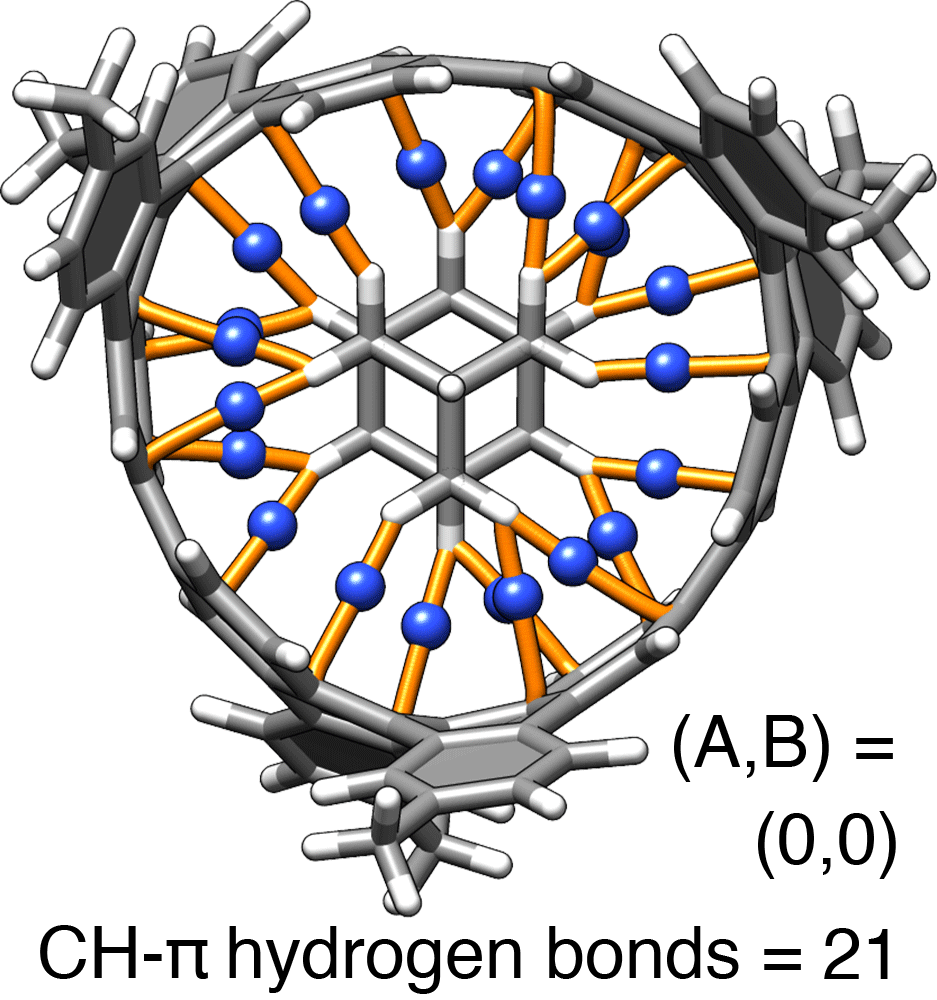

Supplement: Supplementary file 3 — Supplementary Movie 1 [file 41467_2021_25358_MOESM3_ESM.gif]

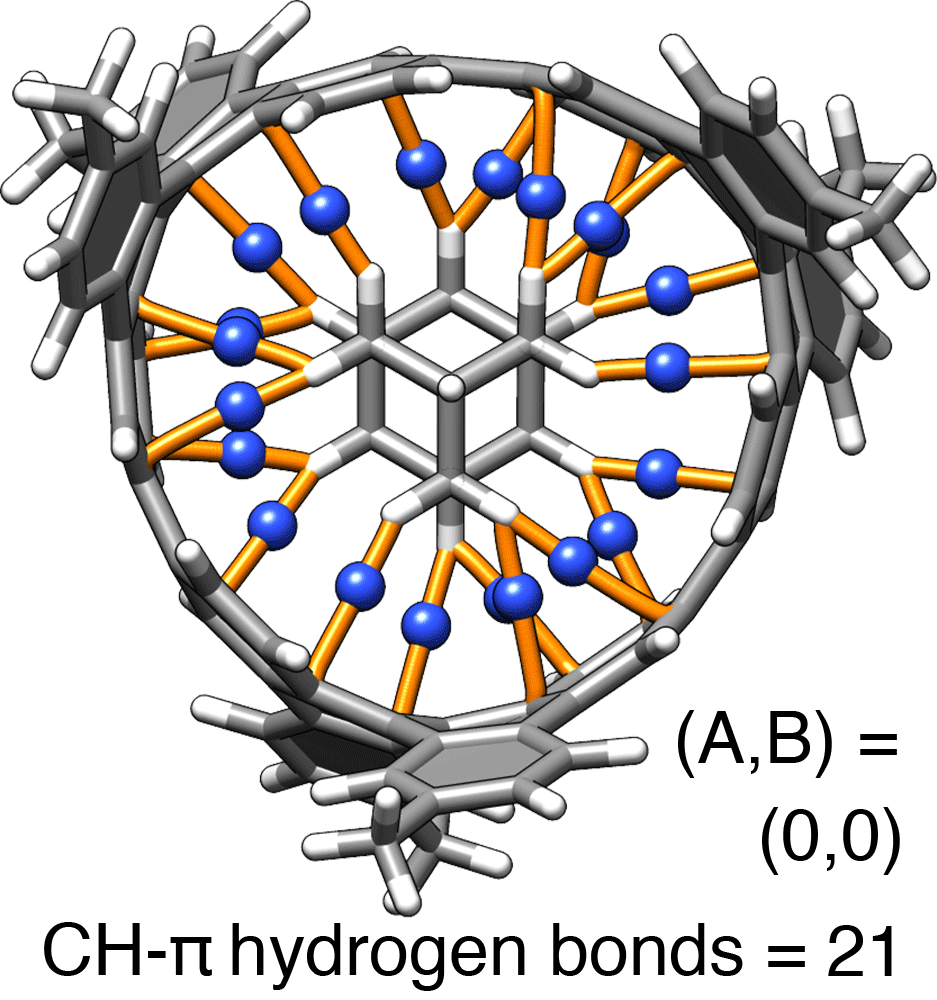

Supplement: Supplementary file 4 — Supplementary Movie 2 [file 41467_2021_25358_MOESM4_ESM.gif]
